# Overexpression of sulfide:quinone reductase (SQR) in *Acidithiobacillus ferrooxidans* enhances sulfur, pyrite, and pyrrhotite oxidation

**DOI:** 10.1128/aem.00170-25

**Published:** 2025-03-25

**Authors:** Heejung Jung, Yuta Inaba, Scott Banta

**Affiliations:** 1 Department of Chemical Engineering, Columbia University 207090 , New York, New York, USA; Colorado School of Mines, Golden, Colorado, USA

**Keywords:** *Acidithiobacillus ferrooxidans*, hydrogen sulfide, sulfur oxidation, sulfide:quinone reductase (SQR), pyrite, pyrrhotite

## Abstract

Hydrogen sulfide is produced during the dissolution of some sulfidic minerals and during the microbial metabolism of reduced sulfur compounds. The sulfide:quinone reductase (SQR) enzyme is able to oxidize H_2_S, and the bioleaching cells *Acidithiobacillus ferrooxidans* have two SQR genes, only one of which has been characterized. We cloned and overexpressed the two SQR genes in *A. ferrooxidans* and show that they both have SQR activity. Both AFE_0267 and AFE_1792 are active under anaerobic conditions, but only AFE_1792 is active under aerobic conditions. The effect of the SQR overexpression and the expression of related genes on sulfur metabolism was investigated. The overexpression of SQR improved cell growth and sulfur oxidation, suggesting enhanced SQR activity led to a reduction in H_2_S toxicity as well as providing additional energy through H_2_S oxidation. Additionally, the impact on the oxidation of pyrite and pyrrhotite was investigated. The rate of oxidation of pyrite by the engineered cells was enhanced, and, furthermore, the rate of pyrrhotite oxidation was more than doubled.

**IMPORTANCE:**

H_2_S is a toxic sulfur intermediate, and the SQR enzyme has evolved to oxidize H_2_S in *A. ferrooxidans*. In addition to detoxification, H_2_S oxidation provides energy, and overexpression of SQR enhanced aerobic and anaerobic growth on sulfur. The SQR overexpression also enhanced pyrite and pyrrhotite oxidation, which may facilitate the pyrometallurgical processing of a number of critical materials including copper, nickel, and the platinum group metals.

## INTRODUCTION

Sulfur has redox states ranging from −2 in sulfide to +6 in sulfate, and these compounds can serve as electron donors or acceptors that support a range of complex metabolic processes, especially in extreme environments (1). Sulfur-reducing microbes can produce hydrogen sulfide (H_2_S) and are well-known for their role in metal corrosion (2). Sulfur-oxidizing microbes have been used for remediation applications including wastewater treatment and desulfurization (3). Sulfur-oxidizing bacteria are also exploited for industrial-scale microbial bioleaching of copper and gold (4); however, sulfur oxidizers are also involved in the generation of acid mine drainage (5). The disulfide pyrite (FeS_2_) and the monosulfide pyrrhotite (Fe_1-x_S) are the two most common sulfide minerals in nature, and the microbial oxidation of these materials can have tremendous environmental impacts (6). The reaction of monosulfides such as pyrrhotite with acid results in the abiotic formation of H_2_S (7).

*Acidithiobacillus ferrooxidans* has emerged as a model bioleaching microorganism (8). These chemolithoautotrophs can oxidize iron or sulfur aerobically, and they can also oxidize sulfur anaerobically when coupled with iron reduction. There has been increasing interest in developing genetic tools for their exploration and development as a synthetic biology platform (9). The genome of the type strain (ATCC 23270) has been determined (10), and this has supported the development of metabolic models (11). With these and other advances, the pathways that support aerobic iron oxidation and anaerobic iron reduction have been largely elucidated (12). However, the sulfur oxidation pathways are far more complex as they involve a number of reduced sulfur intermediate compounds, and thus the specific mechanisms for these reactions are still an area of active investigation (13).

**Fig 1:**
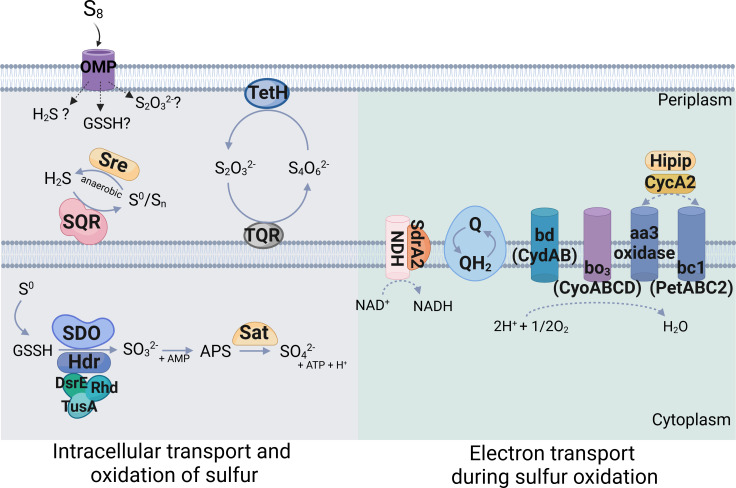
Overall diagram of sulfur oxidation pathways in *A. ferrooxidans*. Abbreviations: OMP, outer-membrane protein; SQR, sulfide:quinone reductase; Sre, sulfur reductase; GSSH, glutathione persulfide; TetH, tetrathionate reductase; TQR, quinone reductase; SDO, sulfur dioxygenase; HDR, heterodisulfide reductase; Sat, ATP sulfurylase; DsrE, TusA, and Rhd, cascade of sulfur transferases; QH_2_, quinone pool; NDH, NADH:quinone oxidoreductase; SdrA2, short-chain dehydrogenase; bd/bo3, terminal oxidases; aa_3_ oxidase, aa_3_-type cytochrome c oxidase; bc1, cytochrome bc1 complex; HiPIP, high-potential iron sulfur protein; CycA2, c4-type cytochrome c. The figure was created by Biorender.com.

To date, it is known that a variety of enzymes, enzyme complexes, and electron carriers are involved in sulfur metabolism of *A. ferrooxidans* (Fig. 1). The exact mechanism for the uptake of extracellular sulfur is not well-understood, although this is known to be mediated by glutathione persulfide (GSSH) (14). Once transported intracellularly, elemental sulfur (S^0^) is converted to sulfate through sulfur dioxygenase (SDO), heterodisulfide reductase (HDR), and ATP sulfurylase (SAT), in which sulfur transferases (DsrE, TusA, and Rhd) are involved (15–17). Other enzymes, including sulfide:quinone reductase (SQR), tetrathionate reductase (TetH), quinone reductase (TQR), and sulfur reductase (Sre), also contribute to the oxidation of reduced sulfur compounds with different oxidation states. The electrons produced during sulfur oxidation are delivered to the quinone pool, which transfers the electrons (i) directly to NADH complex for generating reducing power or (ii) (in)directly to terminal oxidases of bd/bo_3_, and aa_3_ for oxygen reduction (15–17).

Hydrogen sulfide (H_2_S) is a particularly interesting intermediate in the sulfur oxidation process. It has previously been demonstrated that *A. ferrooxidans* can generate H_2_S during the aerobic or anaerobic oxidation of sulfur (18). *A. ferrooxidans* can also use sulfide as an energy source (19–21). The cells express a sulfur reductase (Sre) that generates H_2_S from elemental sulfur, and H_2_S can be generated during the oxidation of minerals including pyrite and especially pyrrhotite (22, 23). The cells can eliminate toxic H_2_S by the sulfide:quinone reductase as this flavoprotein catalyzes the oxidation of H_2_S (and HS^−^ and S^2−^) to elemental sulfur or polysulfide chains. The electrons are transferred from the FAD cofactor of the membrane protein to the ubiquinone or menaquinone pools. Two different SQR genes have been identified in *A. ferrooxidans*. The SQR protein from the AFE_1792 gene (Fig. S1) has been expressed, purified, and biochemically characterized, and an X-ray crystal structure has been elucidated (24–26). The protein product of the AFE_0267 gene has also been annotated as a putative SQR gene (10) with a sequence homologous to AFE_1792 (Fig. S1). When the TetH protein was overexpressed in *A. ferrooxidans*, it was found that the transcription of both AFE_0267 and AFE_1792 were similarly increased with TetH overexpression (27), suggesting that AFE_0267 is also a functional SQR in *A. ferrooxidans*.

In this work, we cloned both the AFE_0267 and AFE_1792 genes and constitutively overexpressed them back in *A. ferrooxidans* ATCC 23270. The impact of the overexpression strains on aerobic and anaerobic sulfur oxidation was evaluated as well as on pyrite and pyrrhotite bioleaching. Overall, these results indicate that both genes encode SQRs and that overexpression of SQR leads to enhanced growth and performance of *A. ferrooxidans*, which may be useful for the development of future biotechnologies.

## RESULTS

### Aerobic and anaerobic sulfur oxidation

Two new *A. ferrooxidans* cell lines were created for the overexpression of the AFE_0267 and AFE_1792 genes, which encode a putative SQR enzyme and a confirmed SQR enzyme, respectively. Digital PCR (dPCR) was used to confirm that the transformed cells had higher transcriptional gene expression compared to the wild-type cells (AF), indicating the successful overexpression of the genes (Fig. S2). These engineered cells were referred to as AF72 and AF73, respectively, and their sulfur oxidation behaviors were compared with the wild-type cells.

**Fig 2:**
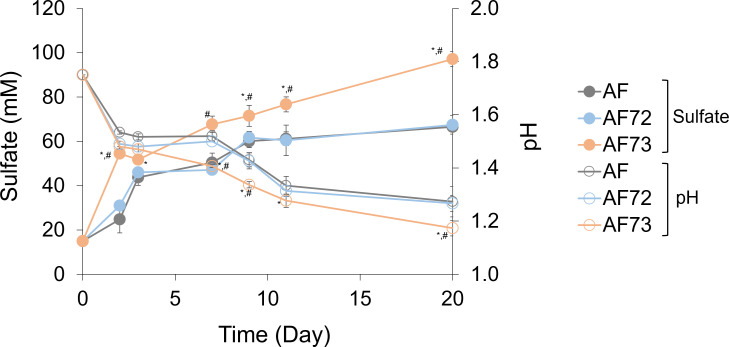
Changes in sulfate concentration and pH during the aerobic sulfur oxidation with the wild-type (AF) and engineered *A. ferrooxidans* with overexpression of two putative SQR proteins of AFE_0267 (AF72) and AFE_1792 (AF73). Standard deviations were obtained from triplicate analyses, and symbols indicate statistical significance (*P* <0.05) compared to AF (*) and between engineered cells (#).

The wild-type and two engineered cell lines were grown with sulfur (0.1%, w/v) under aerobic and anaerobic conditions, and the sulfate production and pH changes were monitored as indicators of sulfur oxidation. Under aerobic conditions, the AF73 cells showed higher sulfate production along with lower final pH in the solutions when compared to the wild-type and AF72 cells, which showed comparable sulfur oxidation behaviors (Fig. 2). The AF73 cells overexpress the AFE_1792 gene, which is a confirmed SQR enzyme, and so these results demonstrate that overexpression of the confirmed SQR gene leads to enhanced aerobic sulfur oxidation behavior.

**Fig 3:**
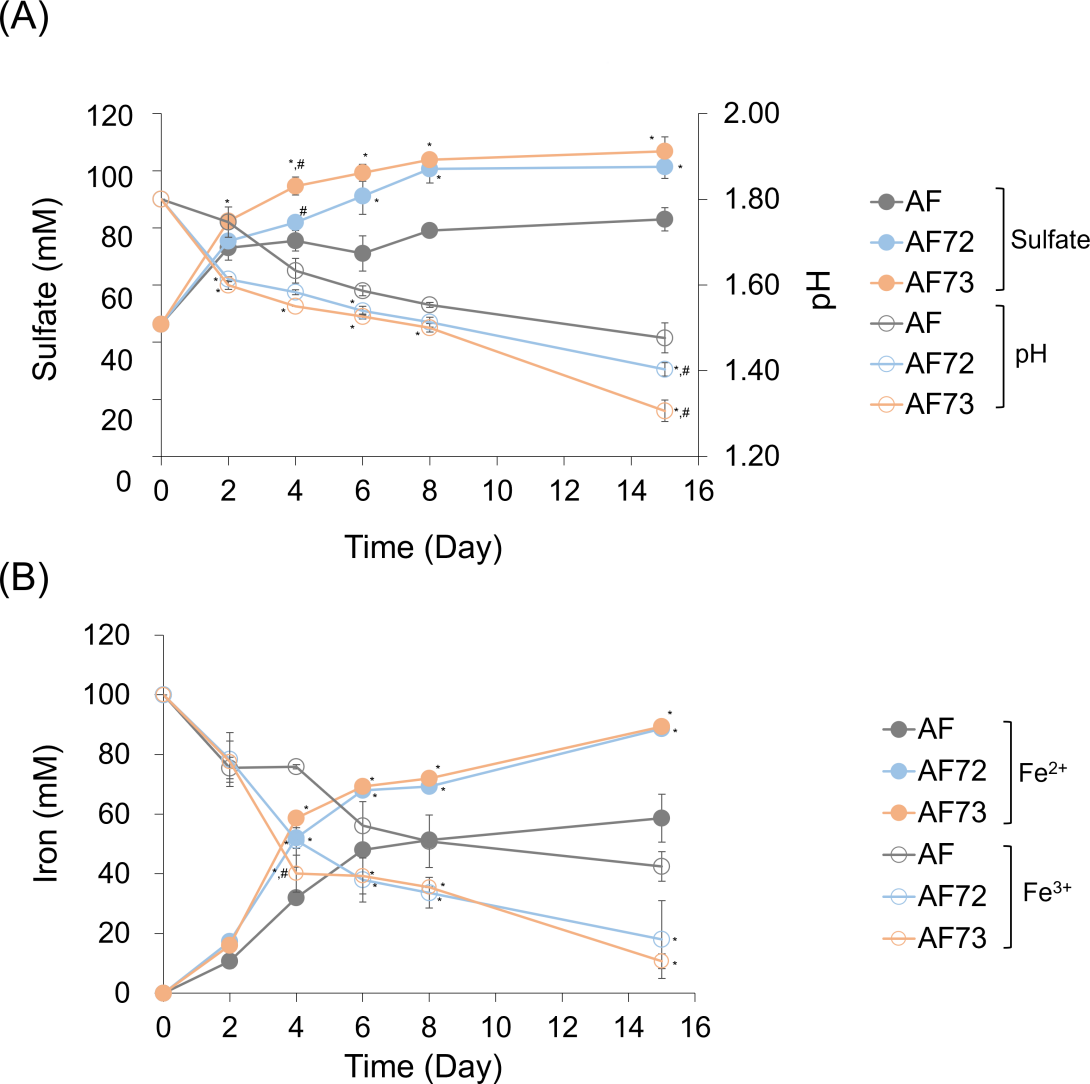
Changes in sulfate concentration and pH (A) and iron (Fe^2+^ and Fe^3+^) concentrations (B) during the anaerobic sulfur oxidation with the wild-type (AF) and engineered *A. ferrooxidans* with overexpression of two putative SQR proteins of AFE_0267 (AF72) and AFE_1792 (AF73). Standard deviations were obtained from triplicate analyses, and symbols indicate statistical significance (*P* <0.05) compared to AF (*) and between engineered cells (#).

For anaerobic growth experiments, Fe (III) was added to enable the coupling of anaerobic sulfur oxidation to iron reduction. Interestingly, both engineered cells showed improved sulfur oxidation along with concomitant iron reduction, as compared to the wild-type cells (Fig. 3). The more rapid iron reduction and sulfur oxidation of the engineered cells were also visually apparent with the clarification of the growth media as compared to the wild-type cells (Fig. S3). These results demonstrate that the AFE_0267 gene also encodes a functional SQR protein which produces a novel phenotype only under anaerobic conditions.

Given the various oxidation states of sulfur (ranging from −2 to +6), different types of reduced sulfur species including sulfite (SO_3_^2−^), thiosulfate (S_2_O_3_^2−^), and polythionates (S_n_O_6_^2−^) can be generated via the complex (non)enzymatic reactions during the sulfur oxidations by *A. ferrooxidans* (9). As the engineered cells enhanced sulfur oxidation under different growth conditions, we measured the changes in the sulfur intermediate species during the growth of the cells to explore the effect of AFE_0267 and AFE_1792 overexpression on the sulfur oxidation pathways. Due to the limited availability and intricate chemical and enzymatic synthesis processes associated with most polythionates, we report the variations of peak areas corresponding to the different sulfur species without quantification, which can be used to provide insight into the changing profiles of the sulfur species during the time course of the experiments. The engineered cells affected the production and consumption of the sulfur intermediates (sulfite, thiosulfate, tri-, tetra-, penta-, and hexathionates) under the different conditions. Under aerobic growth, thiosulfate was the most abundant sulfur species for all *A. ferrooxidans* strains, in terms of peak area (Table S2). Both wild-type and AF72 cells maintained high concentrations of thiosulfate, along with the relatively higher amounts of tri- and tetrathionates. On the other hand, the AF73 cells exhibited a dramatic decrease in thiosulfate over time and relatively lower amounts of sulfite and all the polythionates. By contrast, anaerobic growth of the wild-type cells resulted in lower concentrations of almost all of the sulfur species, especially tetrathionate, although relatively higher levels of the longer chain hexathionate were observed. Under anaerobic conditions, the engineered cells both accumulated more sulfite and hexathionate, as compared to the wild-type cells, while most of the other sulfur species were similar to the wild-type cells.

### Gene expressions under aerobic and anaerobic sulfur oxidations

**Fig 4:**
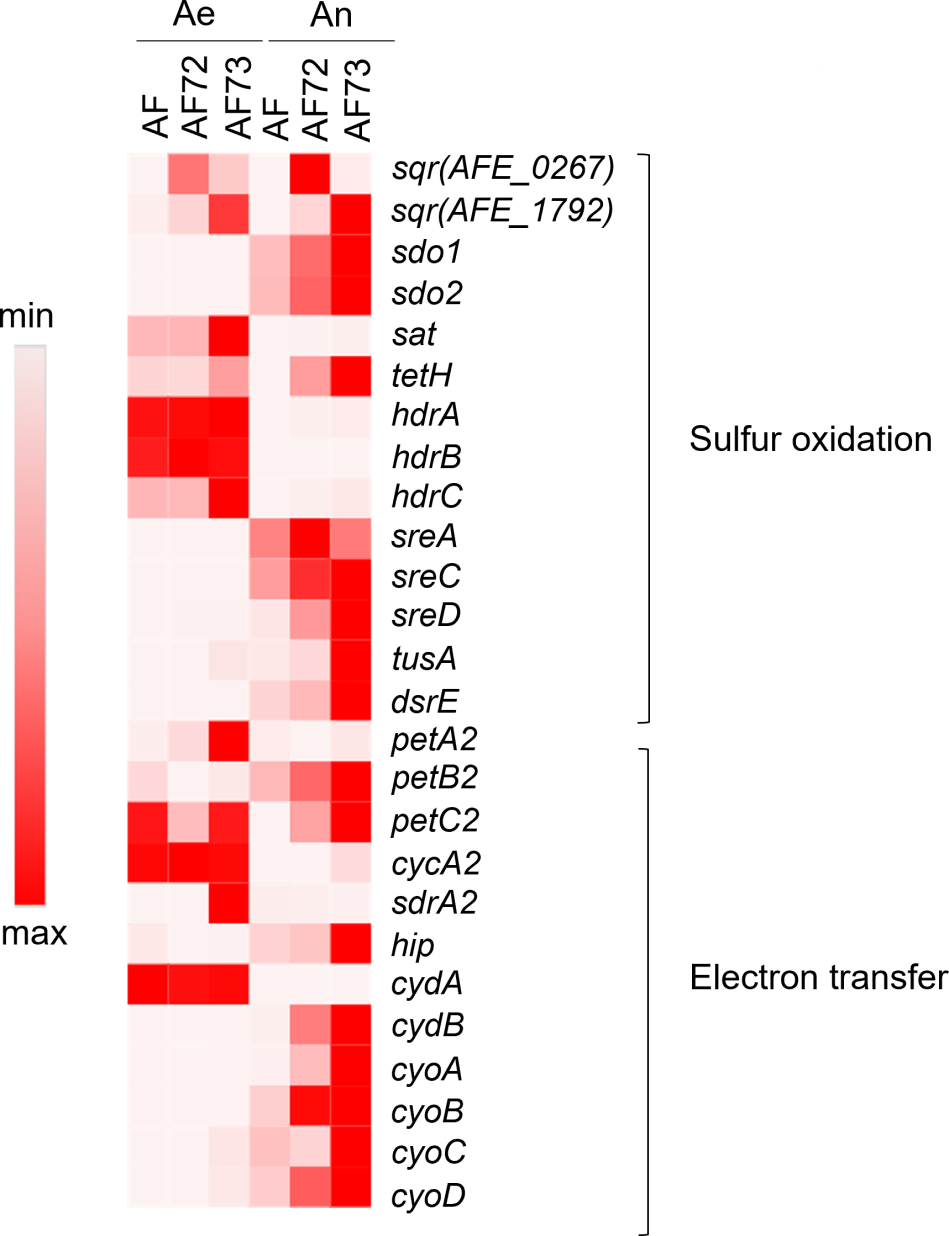
Heatmap distribution of the transcriptional expression of genes encoding proteins responsible for sulfur oxidation, sulfur carrier, and electron transfer of *A. ferrooxidans*, from both aerobic (Ae) and anaerobic (An) sulfur oxidations with the wild-type (AF) and engineered *A. ferrooxidans* with overexpression of two putative SQR proteins of AFE_0267 (AF72) and AFE_1792 (AF73). The relative abundance of gene expressions is presented with the color gradient.

A variety of enzymes, enzyme complexes, and electron carriers are involved in sulfur metabolism of *A. ferrooxidans* (Fig. 1). As enhanced sulfur oxidation by the engineered cells under different growth conditions was observed, the transcriptional expressions of the genes involved in sulfur oxidation pathways were analyzed (Fig. 4). Genes directly involved in sulfur oxidation (*sqr*, *sdo*, *sat*, *tetH*, *hdrABC*, and *sreABCD*), sulfur carriers (*tusA* and *dsrE*), and electron transfer (*petABC2*, *cycA2*, *hip*, *cycA2*, and *sdrA2* [encoding electron transport chain-associated *pet*II operon], *cydAB* [encoding bd oxidase], and *cyoABCD* [encoding bo_3_ oxidase]) were targeted. The transcriptional expressions of targeted genes varied among the cells and between the different growth conditions. Several genes were expressed at higher levels in the wild-type cells either aerobically (*sat*, *hdrABC*, *petBC2*, *cycA2*, and *cydA*) or anaerobically (*sdo12*, *sreAC*, *dsrE*, *petB2*, *hip*, *cyoBCD*). The expression of the *sreB* gene was not detected under any conditions.

As was observed in the growth experiments (Fig. 2), similar trends were seen in the selected gene expression patterns for the AF72 cells and the wild-type cells under aerobic conditions, while the AF73 cells showed an upregulation of several genes (*sat*, *tetH*, *hdrC*, *petA2*, and *sdrA2*). Under anaerobic conditions, the gene expression patterns in AF72 and AF73 were more similar, as compared to the wild-type cells, which was also consistent with the observed growth experiments (Fig. 3). The engineered strains showed increased expression of genes related to sulfur oxidation (*sdo12*, tetH, and *sreCD*), sulfur carriers (*tusA* and *dsrE*), and electron transfer (*petBC2*, *hip*, *cydB*, and *cyoABCD*) as compared to the wild-type cells.

### Effects of improved sulfur oxidation on bioleaching

**Fig 5:**
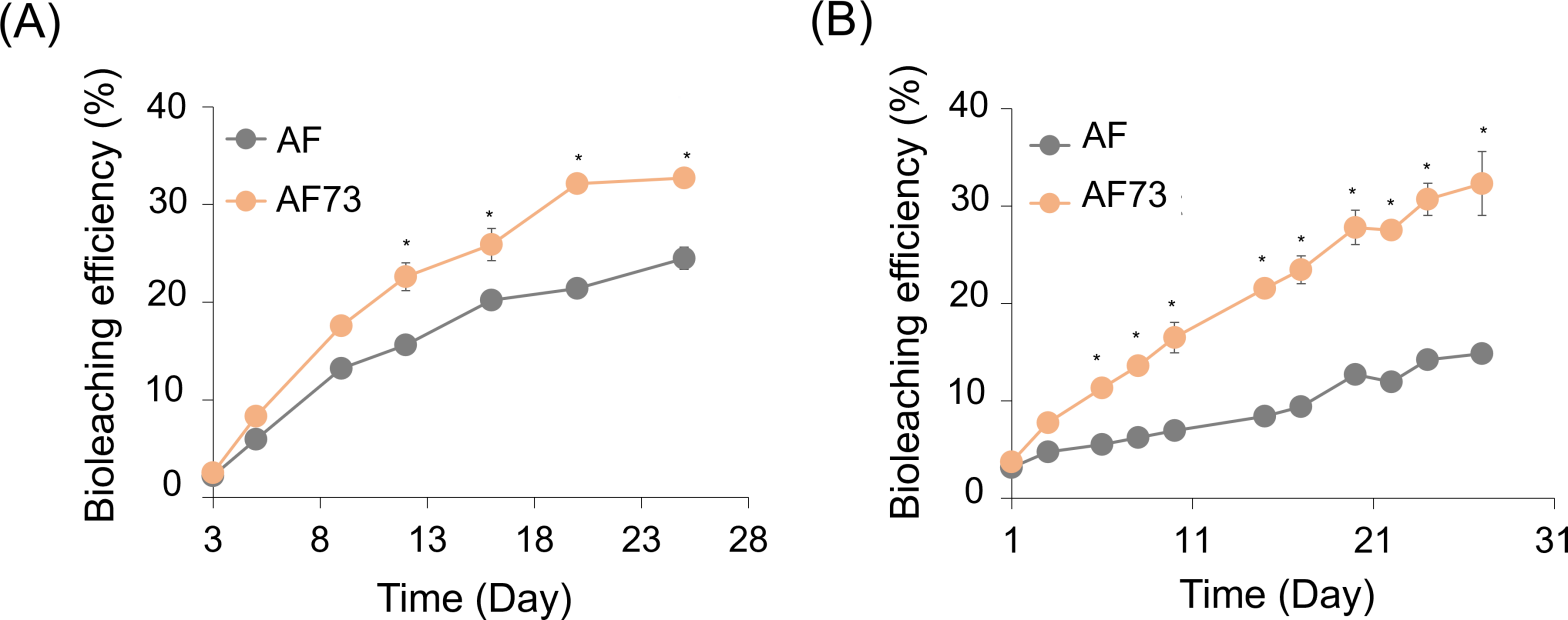
Profiles of aerobic bioleaching efficiency, in terms of iron recovery, of pyrite (A) and pyrrhotite (B) with the wild-type (AF) and engineered *A. ferrooxidans* with overexpression of putative SQR protein of AFE_1792 (AF73). Standard deviations were obtained from triplicate analyses, and the symbol (*) indicates statistical significance (*P* <0.05) between different conditions.

Given that the overexpression of AFE_0267 (AF72) and AFE_1792 (AF73) enhanced sulfur oxidation during the growth of *A. ferrooxidans*, we further explored the potential of improving bioleaching of sulfidic minerals by the engineered cells. Pyrite (FeS_2_) and pyrrhotite (Fe_1-x_S) were chosen for bioleaching as they are important iron sulfide minerals. Considering that the AF73 cells showed better sulfur oxidation than the AF72 cells regardless of growth conditions (aerobic and anaerobic), only the AF73 cells were used for the bioleaching experiments. The engineered cells showed higher bioleaching efficiencies for both pyrite and pyrrhotite, compared to the type cells (Fig. 5). Final bioleaching efficiencies of ~33% for both pyrite and pyrrhotite were achieved by the engineered cells, which were 1.3- and 2.2-fold higher than the wild-type cells, respectively. These indicate that enhanced sulfur oxidations of the AF73 cells also enabled better dissolution of iron sulfides, especially for the monosulfide pyrrhotite which produces H_2_S during oxidation and for which oxidation is often hindered by a rapid buildup of sulfur passivation layers on the surface (6, 28).

## DISCUSSION

H_2_S is the most reduced form of sulfur and is well known for its biological toxicity. However, the microbial metabolism of sulfur, reduced sulfur species, and sulfide minerals can lead to the production of H_2_S, necessitating the evolution of enzymes for its removal. SQR enzymes enable oxidation of H_2_S, and sulfur-oxidizing acidophiles, including *A. ferrooxidans*, are able to oxidize H_2_S aerobically or anaerobically. The SQR enzyme encoded by the AFE_1792 gene has been structurally and biochemically characterized (24–26). We found that the overexpression of the AFE_1792 SQR enhances *A. ferrooxidans* both aerobic (Fig. 2) and anaerobic (Fig. 3) sulfur oxidation and the oxidation of pyrite and pyrrhotite (Fig. 5). We also found that AFE_0267 encodes a gene product with alternative SQR activity that seems to be active only under anaerobic conditions (Fig. 3). Therefore, *A. ferrooxidans* has two SQR genes with different functional activities.

The mechanisms of sulfur oxidation by *A. ferrooxidans* are still being investigated; however, there are several mechanisms by which H_2_S can be generated. A dominant mechanism can occur as extracellular octasulfur (S_8_) is transported across the outer membrane by reaction with the thiol group of an outer membrane protein, generating GSSH, as well as H_2_S. Once in the periplasm, SQR can oxidize the H_2_S to elemental sulfur, which provides a mechanism for elemental sulfur transport into the cell. Elemental sulfur can proceed to be oxidized in the cytoplasm to sulfate. The overexpression of SQR in *A. ferrooxidans* leads to enhanced sulfate production, which suggests that either SQR is a rate-limiting step in this process that can be alleviated by SQR gene overexpression or that the cells respond with increased metabolism and upregulation of other sulfur-related genes upon alleviation of H_2_S toxicity.

Under anaerobic conditions, the expression of both SQR genes led to increased sulfur oxidation, and this was coupled with increases in sulfite and hexathionate levels. The increased SQR activity also correlated with an increase in the expression of several genes involved in sulfur oxidation (*sdo*, *sre tusA*, *dsrE*) and electron transfer (*pet2*, *hip*, *cyo*). The results are consistent with a sulfur oxidation pathway that is dominated by GSSH oxidation by sulfur dioxygenase (*sdo*) that produces sulfite, which is further oxidized to sulfate (14). H_2_S generated during the import of S to make GSSH is oxidized by SQR. Sulfur reductase (*sre*) also reduces sulfur to H_2_S and has been previously shown to be expressed only under anaerobic conditions. The *sre* expression along with *tusA* and *dsrE* expression under anaerobic conditions has supported the identification of an indirect mechanism for ferric iron reduction by H_2_S (15, 16). Therefore, the additional SQR activity may reduce H_2_S toxicity and provide additional energy through H_2_S oxidation.

Under aerobic conditions, a high concentration of thiosulfate was observed, and when the AFE_1792 SQR gene was overexpressed, this led to increased sulfate production and reduced thiosulfate accumulation. This coincided with an increase in the expression of other genes involved in elemental sulfur oxidation including *hdrC*, *sat*, *petA2*, and *sdrA2*, while *sdo* expression was unaffected. These results suggest a more dominant role of the heterodisulfide reductase (*hdr*) pathway for GSSH oxidation and that the increased sulfur production from the enhanced SQR activity led to increases in gene expression for the elemental sulfur oxidation reactions. The impact of SQR overexpression on thiosulfate metabolism is less clear, and future studies would be necessary to determine whether SQR overexpression caused a reduction in thiosulfate production or an increase in thiosulfate accumulation.

Thiosulfate is a dominant intermediate in sulfide mineral oxidation. Pyrite is an acid-insoluble disulfide, and oxidation occurs through a thiosulfate mechanism where ferric iron attacks the pyrite and thiosulfate is produced. The thiosulfate is then oxidized through various intermediates to produce sulfate. When the SQR overexpressing cells were used for pyrite oxidation, the leaching efficiency was enhanced 1.3-fold compared to what was observed for the wild-type cells (Fig. 5A). This is consistent with the previous observations where the engineered cells grown with only sulfur produced more sulfate and less thiosulfate, indicating enhanced overall sulfur metabolism.

Pyrrhotite is a monosulfide which can be dissolved in acid, generating H_2_S. In addition, it can also be oxidized via oxygen and ferric iron attack in a polysulfide-based mechanism. This mechanism generates elemental sulfur which can be oxidized by the bacteria. When the SQR overexpressing cells were used for pyrrhotite oxidation, a more dramatic improvement in bioleaching was observed, where the oxidation efficiency more than doubled (2.2-fold) as compared to the wild-type cells (Fig. 5B). The overexpression of SQR in the engineered cells presumably enhanced oxidation of chemically generated H_2_S as well as enhanced the rate of oxidation of the elemental and intermediate sulfur species.

Microbial pyrrhotite oxidation has received greater recent interest as pyrrhotite is not only associated with sulfide minerals bearing copper and gold, but it is also commonly associated with pentlandite (Fe,Ni)_9_S_8_, marmatite (Fe,Zn)S, and platinum group metal deposits (29). Pyrrhotite can affect the recovery of these materials and contribute to SO_2_ emissions during smelting, and pyrrhotite in tailings can lead to significant acid mine drainage challenges (30). Genetically engineered bioleaching strains with enhanced pyrrhotite oxidation capabilities may be useful as we expand the use of biological approaches toward critical metal production.

## MATERIALS AND METHODS

### Experimental materials

Type strain of *A. ferrooxidans* ATCC 23270 was sourced from ATCC. The F2S growth medium consists of 0.8 g/L (NH_4_)_2_SO_4_, 0.1 g/L K_2_HPO_4_, 2.0 g/L MgSO_4_·7H_2_O, 5 mL/L MD-TMS (Trace mineral solution, ATCC), 27.8 g/L FeSO_4_·7H_2_O, 1.92 g/L citric acid, and 1 g/L dispersed sulfur (#S789400; Toronto Research Chemicals, Toronto, ON) and adjusted the final pH to 1.8 by adding sulfuric acid. AFM1 medium (pH 1.8) consisted of 0.8 g/L, (NH_4_)_2_SO_4_; 0.1 g/L, HK_2_PO_4_; 2.0 g/L, MgSO_4_·7H_2_O; 5 mL/L, MD-TMS; and 72 mM, FeSO_4_·7H_2_O, and SM4 medium (pH 5.0) included 0.8  g/L, (NH_4_)_2_SO_4_; 2.0  g/L, MgSO_4_·7H_2_O; 0.1  g/L, K_2_HPO_4_; 0.19  g/L, citric acid; 5  mL/L, MD-TMS; 40  µg/mL, leucine; 19  µg/mL, diaminopimelic acid; 17.9  µg/mL, Fe_2_(SO_4_)_3_; 1  g/L, dispersed sulfur. Basal salt solution had the same composition as the F2S growth medium, except for iron and sulfur. For the growth experiments, aerobic sulfur growth medium (0.1% [w/v] dispersed sulfur in basal salt solution, pH 1.8) and anaerobic sulfur growth medium (0.1% [w/v] dispersed sulfur and 14 mM Fe_2_(SO_4_)_3_ [Fluka, cat# 31235] in basal salt solution, pH 1.8) were used. The bioleaching solution (pH 1.8) consisted of 0.8 g/L, (NH_4_)_2_SO_4_; 0.1 g/L, HK_2_PO_4_; 2.0 g/L, MgSO_4_·7H_2_O; 5 mL/L, MD-TMS; 10 mM, citric acid; and 100 mM, FeSO_4_·7H_2_O. pH 1.8. All media were filtered through a 0.2 µm pore size (Thermo Fisher Scientific, Waltham, MA) prior to use, and sulfur was added to the media after filtration. All chemicals were purchased from Sigma-Aldrich (St. Louis, MO), unless otherwise noted. Pyrite (cat# 778117, Sigma-Aldrich) and pyrrhotite were used for bioleaching experiments. The pyrrhotite concentrate contained 34% Fe, 1.1% Ni, and 0.1% Cu.

### Genetic manipulation

Genomic DNA of *A. ferrooxidans* was extracted using the NucleoSpin Tissue kit (Takara Bio, Mountain View, CA). Two annotated *sqr* genes (AFE_0267 and AFE_1792) were amplified using the primer sets of pYI72 and pYI73, respectively (Table S1), and purified using NucleoSpin Gel and PCR Clean-up kit (Macherey-Nagel, Düren, Germany). Both amplicons were cloned into the empty pJRD215 vector, pYI39 (31), which was digested with BamHI and KpnI, using NEBuilder HiFi DNA Assembly. The constructs were transformed into *E. coli* DH5α and referred to as pYI72 and pYI73 plasmids, respectively. After sequence verification, both plasmids were transformed into *E. coli* S17-1, then conjugally transferred to *A. ferrooxidans* by filter mating protocol, as previously described (32). The obtained transconjugants were cultured in AFM1 medium and continued with screening in SM4 medium under kanamycin (50 mg/mL), for isolation of the strains overexpressing both *sqr* genes. The isolated strain was referred to as AF72 and AF73, respectively. All plasmids and strains used in this study are presented in Table 1. The plasmid maps for pYI72 and pYI73 are provided in Fig. S2.

**TABLE 1:** Bacterial strains and plasmids used in this study

Strain or plasmid	Description	Source or reference
Strains		
*E. coli* DHα	*F´ proA+B+ lacIq ∆(lacZ)M15 zzf::Tn10 (TetR) / fhuA2∆(argF-lacZ)U169 phoA glnV44 Φ80Δ(lacZ)M15 gyrA96 recA1 relA1 endA1 thi-1 hsdR17*	NEB
*E. coli* S17-1 ATCC 470550	*recA pro hsdR* RP4-2-Tc::Mu-Km::Tn7 integrated into the chromosome	ATCC
*A. ferrooxidans* ATCC 23270	Type strain	ATCC
AF72	ATCC 23270 with pYI72	This study
AF73	ATCC 23270 with pYI73	This study
Plasmids		
pYI39	Empty pJRD215 vector with in-frame polyHis-tag	(31)
pYI72	pYI39 with polyHis-tagged *sqr* (AFE_0267) from *A. ferrooxidans*	This study
pYI73	pYI39 with polyHis-tagged *sqr* (AFE_1792) from *A. ferrooxidans*	This study

### Aerobic and anaerobic sulfur oxidations

The wild-type and engineered *A. ferrooxidans* cells were initially grown in F2S growth medium with an initial optical density measured at 600 nm (OD600) of 0.001 (unless otherwise noted), which corresponds to a cell density of 8.3 × 10^6^ cells/mL (33) and were incubated at 30°C and 150 rpm. The grown cells were harvested at stationary phase (120 h) by centrifugation (5,000 *g* for 7 min) following the removal of sulfur and iron precipitates by low-speed centrifugation (3,000 *g* for 10 sec). The cells were repeatedly washed at 17,000 g for 1 min, using basal salt solutions.

Sulfur oxidation experiments were conducted under aerobic and anaerobic conditions. For aerobic experiments, the washed cell pellet (final OD600 of 0.01) of the wild-type and both engineered cells was inoculated into 100 mL of aerobic sulfur growth medium in a 250 mL Erlenmeyer flask. For anaerobic experiments, the same amount of the washed cell pellet was inoculated into 100 mL of anaerobic sulfur growth medium in a 105 mL Wheaton glass serum bottle. The anaerobic cultures were purged with pure nitrogen gas for 10 min and then sealed with rubber stoppers. The variations in sulfate concentration, pH, and/or iron concentrations of the tested conditions were periodically monitored. All experiments were conducted in triplicate.

### Analysis of sulfur intermediates

The abundance of sulfur intermediates during the time course of sulfur oxidations by *A. ferrooxidans* was analyzed by a Shimadzu Prominence UFLC system equipped with SPD-20A UV-VIS Detector (Shimadzu, Japan), using a Gimini C18 column (250 × 4.6 mm, 5 mm) (Phenomenex, USA). Samples were periodically collected from both aerobic and anaerobic sulfur oxidation experiments and filtered through a PVDF syringe filter with 0.22 mm pore size (Celltreat, USA). A mixture (pH 5.0) of acetonitrile and water (20:80, v/v) containing a 6 mM tetrapropyl ammonium hydroxide was used as mobile phase with a flow rate of 0.6 mL/min. The chromatograms were measured at 230 nm and 23°C. Peaks corresponding to thiosulfate, sulfite, and polythionates (S_n_O_6_^−^, *n* = 3, 4, 5, and 6) were determined by comparison with previous literature values (34, 35).

### Quantitative analysis of gene expression

At the end of the sulfur oxidation experiments under aerobic and anaerobic conditions, 1 mL of samples were collected. Total RNA was extracted with RNeasy Mini kit (Qiagen, USA) following the manufacturer’s instructions. Reverse transcription was performed using a QuantiTect Reverse Transcription Kit (Qiagen, USA), following incubation (at 42°C for 30 min) and thermal inactivation (at 95°C for 5 min). The resulting cDNA was used to analyze genes encoding proteins involved in intracellular sulfur transport (*tusA* and *dsrE*), sulfur oxidation (*sqr* [AFE_0267 and AFE_1792], *sdo*, *sat*, *tetH*, *hdrABC*, and *sreABCD*), and electron transfer during sulfur oxidations (*petABC2*, *cycA2*, *sdrA2*, *hip*, *cydAB*, and *cyoABCD*), in *A. ferrooxidans*, using digital polymeric chain reaction (dPCR) with relevant primers (Table S1). The template cDNA was fragmented by either *EcoR*I or *Xba*I, and the dPCR reactions were prepared using the QIAcuity EG PCR Kit (Qiagen, Germany). The dPCR reactions were loaded onto the QIAcuity Nanoplate 8.5K 96-well and carried out in a QIAcuity ONE 2-Plex system (Qiagen, Germany), with thermal cycling as previously described (36). The absolute gene copy number of samples was calculated by Poisson statistics, and the final gene concentration was normalized by cDNA concentrations. The relative abundance of transcriptional gene expressions was visualized using a Morpheus heatmap tool (https://software.broadinstitute.org/morpheus/).

### Bioleaching experiments

The effect of enhanced sulfur oxidation by SQR overexpression on the bioleaching of iron sulfide minerals (pyrite and pyrrhotite) was examined using AF73 and the wild-type cells, considering the AF73 strains showed better sulfur oxidation than the AF72 cells, regardless of growth conditions. Both wild-type and engineered cells were initially grown in F2S and harvested as described above. The washed cell pellet (final OD600 of 0.05 which corresponds to cell density of 6 × 10^6^ cells/mL) was inoculated into 100 mL of bioleaching solution. A pulp density of 1% (w/v) was used for pyrite and pyrrhotite. The leachate was periodically collected, and soluble iron concentrations were measured to determine bioleaching efficiency. All experiments were conducted in triplicate and incubated at 30°C and shaken at 150 rpm. The water evaporation was compensated by adding distilled water, and pH was adjusted below 2 using sulfuric acid throughout the experiments.

### Other analytical methods

OD600 was measured using a GENESYS 10S UV-VIS spectrophotometer. pH was measured by a pH 700 benchtop meter (Oakton, Vernon Hills, IL). The soluble Fe^2+^ concentrations were measured by titration with cerium sulfate using a ferroin indicator, while total soluble iron concentrations were analyzed by atomic absorption spectrometer (iCE 3300, Thermo Fisher Scientific, Waltham, MA). Sulfate concentrations were measured using a barium sulfate turbidimetric method, following the previous method (37, 38).

Statistical significance of all data was evaluated by *P* value <0.05 through a two-way analysis of variance using Excel.

## Data Availability

The nucleotide sequences of pYI72 and pYI73 have been deposited in GenBank under accession numbers PP070537 and PP070538, respectively.

## SUPPLEMENTAL MATERIAL

Supplemental material aem.00170-25-s0001.pdf Figures S1 to S3; Tables S1 and S2.

ASM does not own the copyrights to Supplemental Material that may be linked to, or accessed through, an article. The authors have granted ASM a non-exclusive, world-wide license to publish the Supplemental Material files. Please contact the corresponding author directly for reuse.
