# Supplementary material for: Overexpression of sulfide:quinone reductase (SQR) in Acidithiobacillus ferrooxidans enhances sulfur, pyrite, and pyrrhotite oxidation
Source: Appl Environ Microbiol. 2025 Mar 25;91(4):e00170-25. doi: 10.1128/aem.00170-25 (PMC12016491; doi:10.1128/aem.00170-25)
Supplement: Supplemental material — Figures S1 to S3; Tables S1 and S2. [file aem.00170-25-s0001.pdf]

Supplemental Materials

Overexpression of sulfide:quinone reductase (SQR) in *Acidithiobacillus ferrooxidans* enhances sulfur, pyrite and pyrrhotite oxidation

Heejung Jung, Yuta Inaba, and Scott Banta*

Department of Chemical Engineering, Columbia University, 500 West 120th Street, New York, NY 10027, USA

* Corresponding author. Mailing address: Department of Chemical Engineering, Columbia University, 820 Mudd MC4721, 500 W. 120th St., New York, NY 10027. Phone: (212) 854-7531, Fax: (212) 854-3054. E-mail: sbanta@columbia.edu

Number of pages: 8

Number of tables: 2

Number of figures: 3

```
T-COFFEE, Version_11.00 (Version_11.00)
Cedric Notredame
SCORE=79
*
 BAD AVG GOOD
*
AFE_1792     :  79
AFE_0267     :  79
cons         :   7

AFE_1792     MAHVVILGAGTGGMPAAYEMKEALGSGHEVTLISANDYFOFVPSNPWVGVGWKERDDIAFPIRHYVERKGIH
AFE_0267     MTQVTIIGAGFGGLTAVRHLRRRMPD-AEITVIAPRAEFVYYPSLIWIPTGLRQGENLRIPLDRFFQRRRVQ

cons         *::*.*:*** **:.*. .::. : .  *:*:*:..   * : **  *: .* :: ::: :*: ::.:*: ::


AFE_1792     FIAOSAEOIDAEAQNITLADGNTVHYDYLMIATGPKLAFENVPGSDPHEGPVOSICT-VDHAERAFAEYOAL
AFE_0267     FHQGRVTGLRDGG-RTVITDQGEVRNDALIIASGGR-GIRKLPGIEHS----FAICDGIDAAEN-IRDRLAL

cons         *     .  :   .  . .::* . *: * *:**:* : .:.::** :      :**  :* **. : :   **


AFE_1792     LREPGPIVIGAMA-----GASCFGPAYEYAMIVASDLKKRGMRDKIPSFTFI-TSEPYIGHLGIOGVGDSKG
AFE_0267     -MDKGTIAFGFAGNPLEPTAVRGGPVFELLFGIDTYLRQIDKRGQIELVFFNPMTEP-GNRLGPKAV---EG

cons          : *.*.:*  .     *    **.:*  : : : *:: . *.:*  . *  :**  .:** :.*    :*


AFE_1792     ILTKGLKEEGIEAYTNCKVTKVEDNKMYVTQVDEKGETIKEMVLPVKFGMMIPAFKGVPAVAGVEGL-CNPG
AFE_0267     LLAE-MQRRDIRTHLGHKISGFSVNKVMT----EGGDIAADLIL------FMPGMTG-PDWAADSGLPLSAG

cons         :*::  ::...*.:: . *:: .. **: .    * *:    ::*       ::*.:.* *  *. .**  ..*


AFE_1792     GFVLVDEHORSKKYANIFAAGIAIAIPPVETTPVPTGAPKTGYMIESMVSAAVHNIKADLEGRKGEOTMGTW
AFE_0267     GFFQSDLHCTVPDHPGVFVIGDGGS-----YAGSPDWLPKQGHMADLQAGTAVHNLLLHLQGKAADNTFRS-

cons         **.  * *    .:..:*. * . :     :  *   ** *:* :   ..:****:  .*:*: .::*: :


AFE_1792     NAVCFADMGDRGAAFIALPQLKPRKVDVFAYGRWVHLAKVAFEKYFIRKMKMGVSEPFYEKVLFKMMGITRL
AFE_0267     ELICIVDTLDSGIMVY----RSPNHASILPNSLW-HAAKVAFEWRYLLHYR---------------------

cons         : :*:.*  * *  .    .*.:..::. . * * ******  :: : :


AFE_1792     KEEDTHRKAS
AFE_0267     ----------

cons
```

Figure S1. Multiple sequence alignment of SQR proteins encoded by AFE_1792 and AFE_0267. Alignment produced using T-Coffee.

Table S1. Sequences of DNA primers used in this study.

Primer		Sequence (5’ → 3’)
Cloning		
pYI72	F	GTGATGGTGATGATGGGTACCGCGGTAATGCAACAGATAACG
	R	ATTTCACACAGGAGGTAAAGGATCCATGACCCAAGTGACCATTATC
pYI73	F	ATTTCACACAGGAGGTAAAGGATCCATGGCACATGTGGTAATTTTGG
	R	GTGATGGTGATGATGGGTACCGGAGGCCTTACGATGGGTATC
Quantitative gene expression		
sqr1	F	TTCAATCCGATGACCGAACC
(AFE_0267)	R	GACCAAGATGGGTGCGAATA
sqr2	F	CGGACCGGCTTACGAATATG
(AFE_1792)	R	GTAGGGCTCACTGGTGATAAAG
sdo1	F	AACGGTAACTTACGCATCCC
(AFE_0269)	R	CGTACTAGTGGATGATGACGATTT
sdo2	F	GGTTTATCCGGCCCATGATTA
(AFE_2644)	R	CCAGTTTCAGATCAGCCATGA
sat	F	TCGAAAGCGATGCCGATAC
	R	GAAATAGCCCTCCTGCTTCTT
tetH	F	CAGCGTATCGGCACAATATCTA
	R	CGGTATCGTCCCTTTCCATAC
hdrA	F	TCACCCTTGACCAGCAAAC
	R	TCTACTGGAAGAGTCAGGAAGAG
hdrB	F	GGCCGATTTCCAGGATTATCTC
	R	GGCGGAAGCCATCTTCATATT
hdrC	F	GCGGGCTATAGTCATAGAACTG

	R	ACGCTGAAATGAGGGAGATG
sreA	F	GGGATTATATCGCCCAGAAGAG
	R	AGAGGTGAAGGCAATGGTATG
sreB	F	GATAAGTTCCTCCGCCAGTTT
	R	TTGGCAACCCATGCAGAT
sreC	F	TTCACCGTTTCCGCTTTCT
	R	CATGTCCAGACGCTCATACC
sreD	F	GATCAGCGAACAACCCTATCA
	R	GGTGAACAGCCGGGTATG
tusA	F	GGGATTCTTGGTCTGCTTGG
	R	GGAGTTGACCTCTGGTCAGG
dsrE	F	CTCCGGCGAACTCAAAGAA
	R	CGAAGCAGAAGTACGGATGAT
petA2	F	CTGTGTTGCATACCGCATTAC
	R	ATTACGCGTGCCGAAAGA
petB2	F	CGGAGGCATTCTTCGGATATG
	R	GTAACCCAACCGCCGATAAA
petC2	F	ACCCAGATGGCGCATAAA
	R	CGCTTGGGATCCTGGTAAA
cycA2	F	GGGCAGAAACGCACCTAT
	R	AGTCTGGAAGCCACGTATTG
sdrA2	F	GCACCATACGCCTCCATTAT
	R	GGCGTGTTGGCATACTCTAT
hip	F	GGAAGTACAATATCAGCCACACCC
	R	CATCGGCGTAAACGCTATACAA

cydA	F	CAATCTTTGGCTTGGCTTCC
	R	CGAGGTTTCCATCCCGTATATC
cydB	F	GGGTAGGAAGATCAGACCAAAG
	R	GATGCCTTCCACGGACAA
cyoA	F	GCCGATTCTGTTGCTCAGT
	R	CGACATCCACCAGCAGATAAA
cyoB	F	CGGTTCCGGCCATCAATTA
	R	GAAGACCTTGGGAAACCAGTAG
cyoC	F	AACTGGCGTCATTGGGTATC
	R	GAATCCAGATCAACCCGAAGAA
cyoD	F	CTGATGTTCGTCGCTACCTTT
	R	GGAAGTAGATCTGCGCGATAAT

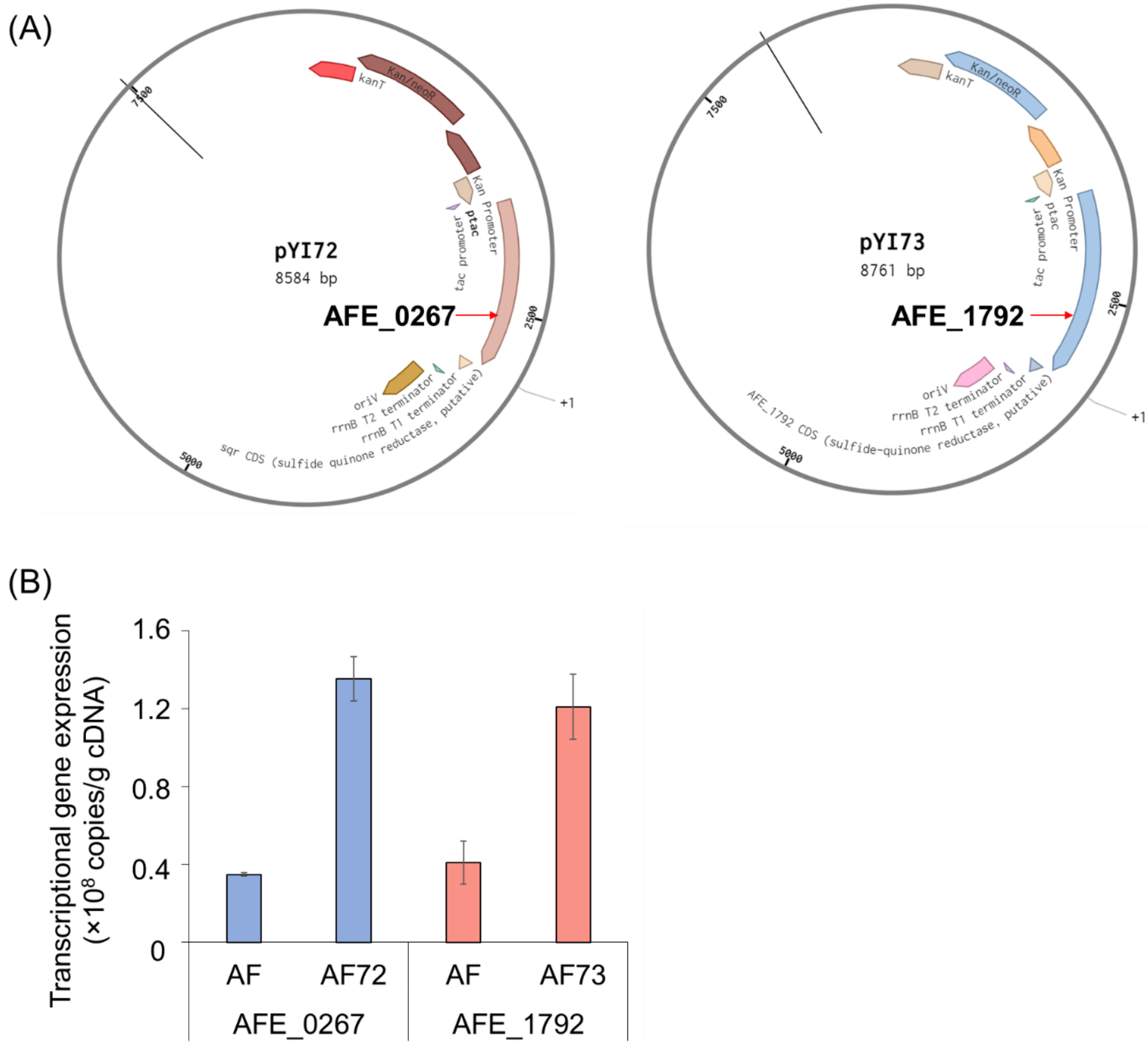

Figure S2. Plasmid maps of pYI72 and pYI73 encoding putative sulfide:quinone reductase (SQR) proteins, AFE_0267 and AFE_1792, respectively, with *tac* promoter (A). The plasmid was created from the pJRD215 vector with *rrnB* terminator and in-frame polyHis sequence. Transcriptional expression of genes encoding AFE_0267 and AFE_1792 in the engineered *A. ferrooxidans* with overexpression of each protein, AF72 and AF73, respectively, compared to the wild type cell (AF). Both wild type and engineered cells for measuring transcriptional gene expression were initially grown in F2S medium.

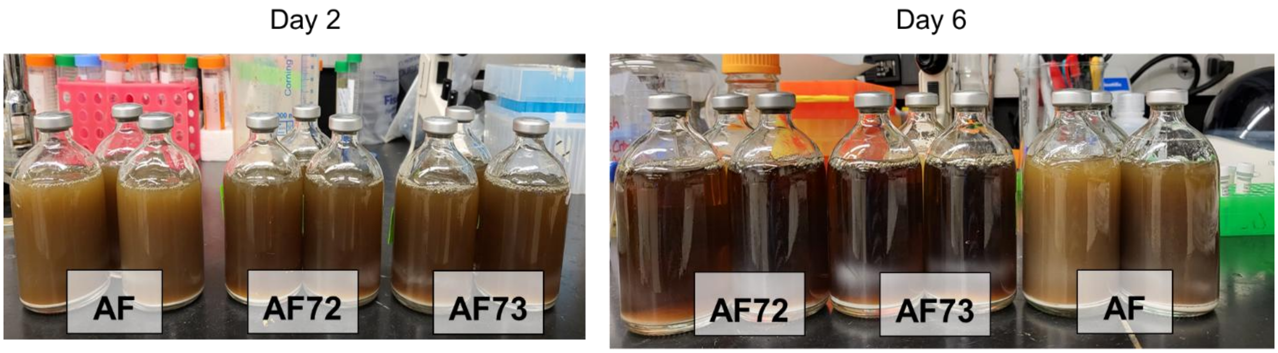

Figure S3. Images of batch cultures of anaerobic sulfur oxidation with Fe^{3+} reduction with the wild type (AF) and engineered *A. ferrooxidans* with overexpression of two putative SQR proteins of AFE_0267 (AF72) and AFE_1792 (AF73), on Day 2 and Day 6.

Table S2. Peak areas of different sulfur intermediates produced during aerobic and anaerobic sulfur oxidations with the wild type (AF) and engineered *A. ferrooxidans* with overexpression of two putative SQR proteins of AFE_0267 (AF72) and AFE_1792 (AF73), analyzed by HPLC.

Aerobic						
**AF**						
Day	SO_3^{2-}	$S_2O_3^{2-}$	$S_3O_6^{2-}$	$S_4O_6^{2-}$	$S_5O_6^{2-}$	$S_6O_6^{2-}$
3	12±1	3600±100	250±20	60±6	5.7±1.1	7.8±1.3
7	19±2	3300±400	150±40	55±6	4.8±1.1	5.0±1.0
9	23±4	3000±600	88±10	60±2	4.1±0.2	3.3±0.5
11	31±3	3100±200	96±6	68±2	5.4±0.4	4.6±0.7
20	23±3	3000±200	86±9	62±4	4.2±0.9	4.6±0.4
**AF72**						
Day	SO_3^{2-}	$S_2O_3^{2-}$	$S_3O_6^{2-}$	$S_4O_6^{2-}$	$S_5O_6^{2-}$	$S_6O_6^{2-}$
3	14±2	3300±300	240±10	77±18	3.6±0.1	6.4±1.0
7	17±5	3000±300	240±20	72±10	1.7±0.3	7.0±1.5
9	20±5	3300±300	200±20	63±11	1.2±0.2	5.9±0.7
11	26±1	3200±400	140±30	68±26	2.9±0.2	4.4±0.9
20	25±1	2900±300	97±5	70±21	3.9±0.1	4.9±0.7
**AF73**						
Day	SO_3^{2-}	$S_2O_3^{2-}$	$S_3O_6^{2-}$	$S_4O_6^{2-}$	$S_5O_6^{2-}$	$S_6O_6^{2-}$
3	3.6±1.5	2500±300	3.0±1.3	0.82±0.04	1.0±0.1	2.3±0.6
7	7.3±1.2	2000±300	2.0±0.1	1.0±0.1	1.5±0.2	5.8±0.4
9	1.3±0.3	1600±300	1.0±0.1	13±3	0.91±0.13	4.3±0.3
11	3.1±0.9	690±280	0.83±0.04	21±2	0.89±0.09	4.8±0.5
20	3.2±0.3	360±90	0.81±0.01	12±1	1.0±1.0	3.7±0.5

Anaerobic						
**AF**						
Day	SO_3^{2-}	$S_2O_3^{2-}$	$S_3O_6^{2-}$	$S_4O_6^{2-}$	$S_5O_6^{2-}$	$S_6O_6^{2-}$
3	6.1±1.2	1.3±0.1	25±3	4.6±0.2	2.4±0.1	17±3
7	18±1	1.0±0.1	9.8±0.4	3.6±0.2	2.3±0.1	22±1
9	13±1	0.87±0.05	8.3±0.3	3.1±0.1	2.1±0.1	34±4
11	15±1	0.80±0.02	8.5±1.0	3.9±1.9	2.1±0.1	31±1
20	10±1	0.85±0.08	8.2±0.3	3.0±0.1	2.1±0.2	33±2
**AF72**						
Day	SO_3^{2-}	$S_2O_3^{2-}$	$S_3O_6^{2-}$	$S_4O_6^{2-}$	$S_5O_6^{2-}$	$S_6O_6^{2-}$
3	5.9±0.4	1.2±0.2	21±3	2.7±0.5	2.1±0.1	25±3
7	16±1	1.0±0.1	21±3	5.6±0.6	2.8±0.3	35±8
9	21±2	0.86±0.16	15±4	5.9±0.9	2.1±0.2	95±10

11	29±3	0.74±0.24	17±1	3.5±0.4	2.1±0.3	150±20
20	36±2	0.94±0.31	8.8±2.4	2.7±0.2	2.2±0.2	120±20
**AF73**						
Day	SO_3^{2-}	$S_2O_3^{2-}$	$S_3O_6^{2-}$	$S_4O_6^{2-}$	$S_5O_6^{2-}$	$S_6O_6^{2-}$
3	8.7±0.3	1.3±0.1	23±1	4.4±0.1	2.5±0.2	39±4
7	22±1	1.1±0.1	30±1	6.4±0.1	2.3±0.1	70±5
9	37±1	1.0±0.1	29±1	13±1	2.2±0.1	85±4
11	49±1	0.88±0.04	16±3	6.3±0.1	2.1±0.1	150±10
20	47±3	0.77±0.03	7.1±0.6	2.1±0.2	2.1±0.1	94±15
